# Preferred Learning Styles among Ophthalmology Residents: An Iranian Sample

**DOI:** 10.18502/jovr.v14i4.5457

**Published:** 2019-10-24

**Authors:** Samira Hassanzadeh, Hossein Karimi Moonaghi, Akbar Derakhshan, Seyed Masoud Hosseini, Ali Taghipour

**Affiliations:** ^1^Paramedical College, Mashhad University of Medical Sciences, Mashhad, Iran; ^2^Evidence- Based Caring Research Center, Department of Medical Surgical Nursing, School of Nursing and Midwifery, Mashhad University of Medical Sciences, Mashhad, Iran; ^3^Department of Medical Education, School of Medicine, Mashhad University of Medical Sciences, Mashhad, Iran; ^4^Eye Research Center, Mashhad University of Medical Sciences, Mashhad, Iran; ^5^School of Nursing and Midwifery, Mashhad University of Medical Sciences, Mashhad, Iran; ^6^Social Determinants of Health Research Center, Mashhad University of Medical Sciences, Mashhad, Iran

**Keywords:** learning Style, Resident, Ophthalmology, VARK Inventory, Kolb Inventory, Iran

## Abstract

**Purpose:**

This study was performed to assess the learning styles of a sample of Iranian residents through Kolb's and VARK questionnaires.

**Methods:**

In this descriptive-analytical study, 45 ophthalmology residents of Mashhad University of Medical Sciences were enrolled. Kolb's and VARK questionnaires were provided, and residents were oriented and guided on how to complete them.

**Results:**

Forty-three out of the forty-five ophthalmology residents completed the questionnaire (95.5% response rate). The preferred learning style among ophthalmology residents was assimilative (51.2%), followed by convergent (37.2%), accommodative (7.7%), and divergent (4.7%), based on Kolb's questionnaire. According to the results of the VARK questionnaire, most ophthalmology residents were auditory learners (34.9%), followed by multimodal learners (30.2%). In addition, there was no significant relation between genders, stage of residency, and Kolb's and VARK learning styles (*P* > 0.05 for all).

**Conclusion:**

The most preferred learning styles of ophthalmology residents were assimilative and auditory. Considering the dominant learning styles of learners and incorporating various teaching methods are recommended to enhance the learning among residents.

##  INTRODUCTION

**Figure 1:**
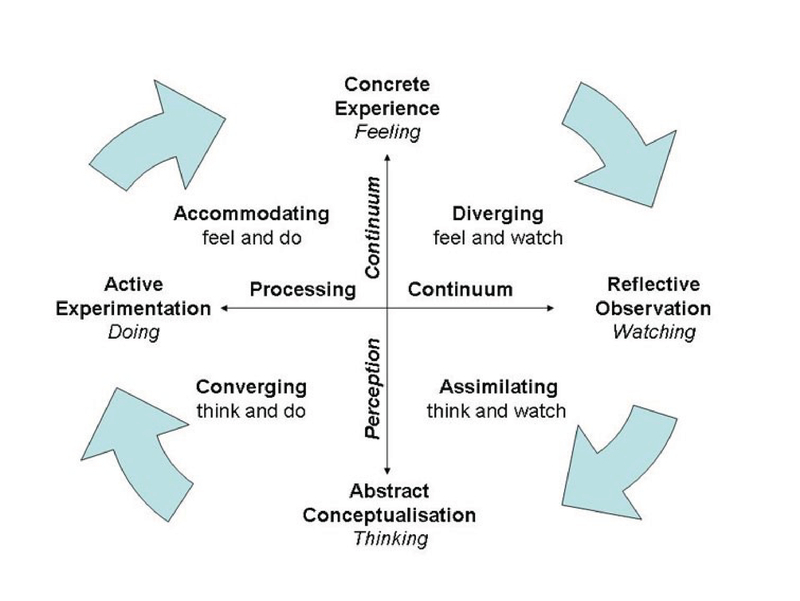
The experiential Kolb's learning cycle.

Learning is a complex process that can be affected by many factors, such as intelligence, motivation, environment, and learning style.^[1]^ Each student uses their unique learning style to learn and process information in different ways.^[2]^ Successful teaching in the field of medical sciences requires knowing a variety of students' learning styles to meet the needs of learners.^[3,4,5,6,7]^ To have a framework to systematically organize the learning style constructs, Curry, in 1983, proposed the “onion ring model.” In this multilayer model, cognitive personality style is in the innermost layer, followed by information processing, social interaction, and instructional preferences in the outermost layer.^[8]^ Curry states that the outermost layer seems to be the less stable one and is likely to be influenced by external factors more than the other layers. Kolb's learning styles questionnaire is a well-known device to assess learning styles of learners,^[9]^ which can then assess the information processing style of an individual. According to Kolb's theory, learning takes place in a cycle comprising four stages [Figure 1]: (1) Facing a topic or learning material (Concrete Experience); (2) Observing and reflecting on it (Reflective Observation); (3) Thinking and conceptualizing (Abstract Conceptualization); and (4) Experiencing (Active Experimentation).^[10]^ However, since learners have different preferences and interests, their function is not the same at each stage of the cycle. This is why different learners choose different careers or fields of study.^[11]^ Kolb divided learners into four learning style groups, based on their preferred action at different stages of Kolb's cycle: diverging, converging, accommodating, and assimilating learners.^[12]^

Students with a divergent learning style use objective experience to learn. They learn more through group discussions and brainstorming. Converging learners use abstract thinking along with an active experience of information to decide how to solve a problem and find a solution. Accommodating learners learn from the combination of objective experience and active experimentation and new experiences. For students with assimilative learning style, abstract thinking and observational evidence have a significant impact on their rational understanding.^[1]^

Mills and Fleming, in 2004, introduced the VARK learning styles questionnaire, which is an abbreviation of four learning styles: visual, auditory–aural, read–write, and kinesthetic. It tests the outer layer of Curry's onion ring model, which is “instructional preferences.”^[8]^ A kinesthetic learner prefers to experience, move, touch, and do things. Also, learners who use two or more of these learning preferences equally are called multimodal learners.^[13]^ A good learner is one who uses all of these functions to learn efficiently, but most of the time, learners show a dominant learning style, which is detectable through this questionnaire. The VARK questionnaire shows how people use their dominant learning style in an environment, while Kolb's questionnaire assesses how the students learn.

Previous studies have shown that most Iranian medical students are converging learners.^[14,15]^ However, our knowledge about our residents' learning styles in different fields is very low. Studies show that knowing the learners' dominant learning styles helps educational systems select teaching methods tailored to those styles.^[10]^ Hence, being familiar with the residents' learning styles at different levels and in different fields of study and various educational settings is of great importance. In this study, we assessed the learning styles of a sample of Iranian residents through Kolb's and VARK questionnaires to evaluate these styles in different aspects and find a possible relation between the results of the two questionnaires.

##  METHODS

Forty-five ophthalmology residents from the Mashhad University of Medical Sciences were enrolled in this descriptive-analytical study in 2017–2018. The study was performed at the Khatam Al-Anbia Eye Hospital, Mashhad, Iran. Informed consent was obtained from all participants, and ethical considerations, such as confidentiality of the participants' personal information, were considered. We also secured an approval from our institutional ethics committee (code: 960226, IR.MUMS.fm.REC.1396.333).

The residents first got acquainted with the questionnaires and then found out how to complete them. Each participant was given the opportunity to familiarize themselves with the learning styles after the study, and the results of their analysis of responses were provided in case of interest.

The validity and reliability of the Persian version of Kolb's and VARK questionnaires have been evaluated in several studies.^[16,17,18,19,20,21,22]^ Kolb's questionnaire consists of 12 statements, each with four answers. Participants must rank the answers from one to four according to their learning preferences. Each statement indicates one element of Kolb's learning cycle. The sum of scores for each answer is plotted on a chart, and the participant's tendency toward a specific learning style is determined. The VARK questionnaire has 16 questions for which the participant must choose one or more of the four answers according to his/her performance in that situation. There is one point for each answer, and the sum of its score is compared with that of the others. Since each question option indicates one VARK learning style (visual, auditory, read and write, or kinesthetic), the highest score in each field reveals the participant's dominant learning style. When the sum of scores in two or more answers is the same, the learner is considered a multimodal one. In this study, we tried to find a connection between the results of the two questionnaires. Also, the relationship between gender, stage of residency, and preferred learning style of the participants was assessed.

##  RESULTS

Forty-three out of the forty-five ophthalmology residents completed the questionnaires correctly (response rate 95.5%). The age of the participants in the study was 30.23 ± 3.40 years (range: 26–46 years), and 13 (30.2%) were female and 30 (69.8%) were male. The dominant Kolb's learning style among the participants was assimilative (51.2%), followed by convergent (37.2%), accommodative, (7.0%) and divergent (4.60%).

**Figure 2:**
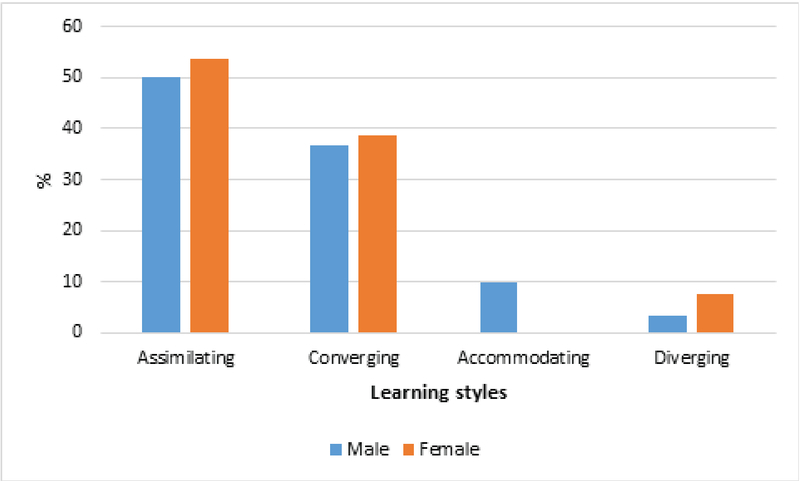
Frequency distribution of Kolb’s learning styles according to gender.

The Chi-square test showed no significant relation between learning style and gender (*P* = 0.636) [Figure 2]. Also, according to the χ${}^{2}$ test, there was no significant relation between learning styles and year of residency (*P* = 0.577) [Table 1].

**Table 1:** Distribution of Kolb's learning styles (in %) according to the year of residency


	**Assimilative**	**Convergent**	**Accommodative**	**Divergent**
**1st year**	50.0	20.0	20.0	10.0
**2nd year**	61.5	38.5	0.0	0.0
**3th year **	50.0	50.0	0.0	0.0
**4th year**	41.7	41.7	8.3	8.3
	
	

According to the VARK questionnaire, most were auditory learners (34.9%), while the rest were multimodal (30.2%), visual (18.6%), read and write (9.0%), and kinesthetic ones (7.0%). Among the multimodal learners, 61.5% were trimodal and 38.5% were bimodal.

**Figure 3:**
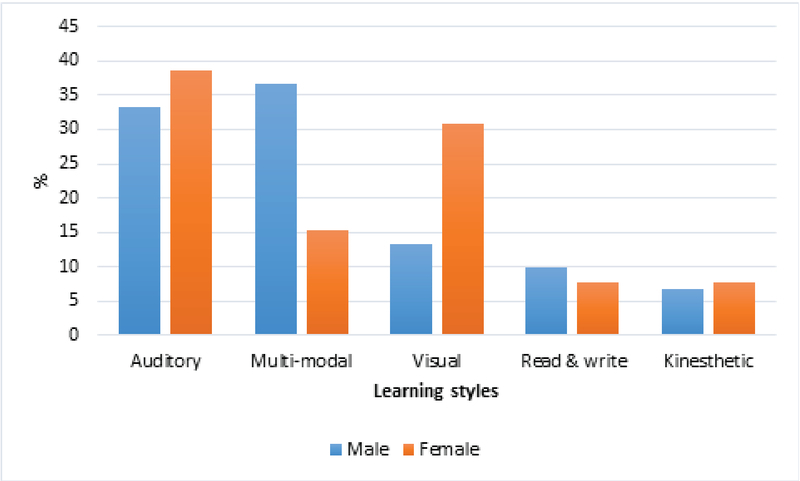
Distribution of VARK’s learning styles according to gender.

Our results showed no significant relation between the preferred learning style obtained through the VARK questionnaire and the gender of the residents (*P* = 0.562) [Figure 3]. We did not also find any significant relation between VARK learning styles and the stage of residency of participants (*P* = 0.728) [Table 2].

**Table 2:** Distribution of VARK learning styles (in %) according to the year of residency


	**Auditory**	**Multimodal**	**Visual**	**Read & Write**	**Kinesthetic**
**1st year**	50.0	30.0	0.0	10.0	10.0
**2nd year**	23.0	38.5	15.4	15.4	7.7
**3th year**	37.5	12.5	37.5	0.0	12.5
**4th year**	33.3	33.3	25.0	8.4	0.0
	
	

##  DISCUSSION

Although several studies have been performed on learning styles of medical students in our country, the research on experiential learning styles of residents in different fields of medicine is scarce. According to our results, the preferred learning style among ophthalmology residents is assimilative. Previous studies showed that the dominant Kolb's learning styles among Iranian medical students were convergent^[22,23,24,25,26]^ and assimilative.^[1,10,21,27,28]^ Studies on non-Iranian residents in various specialties, especially in more practical residency fields such as surgical programs showed that most learners were converging ones.^[14,29,30,31,32,33,34]^ Also, in those studies, more ophthalmology residents were converging learners.^[35,36]^ To our knowledge, the only study to assess the learning styles of Iranian residents was conducted by Ghajarzadeh and her colleagues at the Tehran University of Medical Sciences in 2012,^[1]^ in which 73 residents from seven specialties—pediatrics, general surgery, psychiatry, emergency medicine, internal medicine, radiology, and ophthalmology—were evaluated. According to their results, the dominant learning styles of all residents except for internists were assimilative and convergent. Internal medicine residents' preferred learning style was convergent. The preferred learning style among ophthalmology residents, according to Kolb's questionnaire, was assimilative, which is in concordance with our findings.

According to Kolb,^[37]^ converging learners think and act more. Convergent learning style tends to be the dominant one in careers like medicine and engineering. Such learners are good at professions requiring technical, decision-making, and problem-solving skills. They prefer to experience ideas, practical learning, and simulations. On the other hand, assimilating learners think and watch more. They are oriented to mathematics and physical sciences, and good at planning and research, data gathering, and analysis. Moreover, in learning situations, they prefer lectures and reading and thinking about analytical models.^[37]^

Kolb's studies show that those majoring in applied areas such as medicine are mostly converging learners,^[37]^ which contradicts the results of our study. However, some experts have criticized Kolb's theory for not paying enough attention to different conditions and cultures and the fact that the questionnaire has been examined in a few Western societies.^[12]^

A review study carried out by Hashemi and his colleagues in 2014 showed a higher cataract surgery complication rate by Iranian ophthalmology residents as compared to their counterparts from other countries.^[38]^ Phacoemulsification is one of the most common cataract procedures performed by ophthalmology residents. They found that while in some countries such as USA, the rate of vitreous loss had reached zero from 2001 to 2011, the same by Iranian ophthalmology residents was 10.2%. They stated that as, nowadays, the approach is focused on active learning and problem solving, our educational residency program needs to be changed, with more emphasis on practicality, and orient more learners to be converging ones.^[38]^ Previous studies have shown that learning style can change during an educational course.^[11]^ Also, changes can be introduced to learning styles through practical learning.^[39]^ However, the aim of the educational system is not changing teaching methods according to learners' learning styles. Instead, to achieve better learning outcomes, all students should be given the opportunity to apply all learning preferences according to Kolb's learning cycle during their educational program, and the key to reach this goal is knowing students' educational needs.

Based on the results of the VARK questionnaire, most ophthalmology residents are auditory learners. Even among the multimodal learners, the aural–auditory mode is one of the preferences. Most studies conducted on Iranian medical students have revealed that auditory, followed by multimodal are the dominant learning preferences, which is in agreement with our results.^[16,18,19,20,40,41,42,43]^ However, previous reports also showed that most non-Iranian residents are multimodal learners.^[44]^

An aural–auditory student learns best when they listen to lectures or presentations, discuss concepts, and watch tutorials, while a multimodal learner uses two or more ways of acquiring information to learn. According to the teacher-centered, lecture-based educational system in our country, it can be expected that the auditory learning style will be predominantly strengthened in our students. On the other hand, our ophthalmology residents are mostly assimilating learners. They learn best when they listen to lectures, watch things, and think about ideas; so, it seems logical if they dominantly prefer the auditory learning style.

According to our findings, with both administered questionnaires, there is no significant relation between gender and the preferred learning style among ophthalmology residents. Results of previous studies are controversial.^[16,20,40,41,42]^ The reason can be the effect of cultural differences on learning styles.^[45]^ Kolb believes that in some cultures, where gender roles are less pervasive, men and women's experiences are more homogenous.^[37]^ Considering Kolb's findings, the results of different studies carried out in different cultures can be different, so that, in some of them, there is a significant difference between men and women's preferred learning styles.

Our results using Kolb's and VARK questionnaires show no significant difference between residents in different years of residency and their dominant learning style. Some reports state that there is a change in the learning style of medical students during the course of the undergraduate program^[11]^ and then the residency program.^[31]^ As our study was performed on residents in the same learning environment and same educational program, we can expect that they do not have significantly different preferred learning styles.

Although it is believed that learning styles can be flexible, based on Curry's theory, the inner layer of the onion ring model (information processing), which is assessed by Kolb's questionnaire, is likely to be more stable than the outermost layer (instructional preferences), which is evaluated using the VARK questionnaire.^[8]^ However, in concordance with the results of Mitchell et al,^[8]^ we did not find a significant change in learning styles of residents in different years of residency with both tools. It means that flexibility and changes in learning styles seem to be equal in both information processing and instructional preference modalities.

Our results show that most of our ophthalmology residents are assimilating learners for whom information processing is better done with abstract thinking and observational evidence. Also, their preferred instructional modality is auditory or oral, which means they learn better when they listen to lectures or presentations, discuss concepts, and watch tutorials.

Finally, for researchers interested in similar works, we suggest recruiting residents from one surgical and one medical specialty, and comparing them to ophthalmology residents. Performing more studies and comparing residents in different settings and specialties could be helpful in enhancing our knowledge about the variables that affect residents' learning preferences.

In summary, researchers believe that students adapt to the curriculum.^[11]^ Hence, we can change a teacher-centered, lecture-based model to a student-centered, problem- and practical-based one. Considering the predominant learning style of our residents—assimilative—and due to the practical nature of the field of ophthalmology, we should evaluate the curriculum and orient the learner's learning style toward the goals and needs of the educational program. In addition, as we have different learners, various teaching methods should be incorporated to enhance learning outcomes of the residents.

##  Financial Support and Sponsorship

This study has been supported by a research grant from the Vice-chancellor for Research at the Mashhad University of Medical Sciences (No: 960226).

##  Conflicts of Interest

There are no conflicts of interest.

##  Acknowledgments

This is an approved master thesis (code no: 960226) by the Vice Chancellor for Research (VCR) at Mashhad University of Medical Sciences who supported this study. The authors are thankful to the VCR and all of the ophthalmology residents who contributed to this research despite their lack of time.

## References

[1] Ghajarzadeh M., Adili-Aghdam F. (2012). Learning styles of medical residents of different disciplines in. *Razi J Med Sci*.

[2] ALQahtani D. A., Al-Gahtani S. M. (2014). Assessing learning styles of saudi dental students using kolb's learning style inventory. *Journal of Dental Education*.

[3] Hossein K. M., Fatemeh D., Fatemeh O. S., Katri V.-J., Tahereh B. (2010). Teaching style in clinical nursing education: A qualitative study of Iranian nursing teachers' experiences. *Nurse Education in Practice*.

[4] Karimi-Moonaghi H., Dabaghi F., Oskouei F., VehvilΣinen-Julkunen K. (2009). Learning style in theoretical courses: nursing studentsperceptions and experiences. Iran J Med Educ. *Vehviläinen-Julkunen K*.

[5] Kim R. H., Gilbert T. (2015). Learning style preferences of surgical residency applicants. *Journal of Surgical Research*.

[6] Kim R. H., Gilbert T., Ristig K. (2015). The Effect of Surgical Resident Learning Style Preferences on American Board of Surgery In-Training Examination Scores. *Journal of Surgical Education*.

[7] Kolb D. (1984). *Experiential education: experience as the source of learning and development*.

[8] Mitchell E. K., James S., D’Amore A. (2015). How learning styles and preferences of first-year nursing and midwifery students change. *Australian Journal of Education*.

[9] Kolb DA.

[10] Hosseini S. M., Amery H., Emadzadeh A., Babazadeh S. (2015). Dental Students' Educational Achievement in Relation to Their Learning Styles: A Cross-Sectional Study in Iran. *Global Journal of Health Science*.

[11] Bitran M., Zuniga D., Lafuente M. (2005). *Influence of personality and learning styles in the choice of medical specialty. Rev Med Chile*.

[12] Seif AA. (2016). *An introduction to learning theories*.

[13] Prithishkumar I., Michael S. (2014). Understanding your student: Using the VARK model. *Journal of Postgraduate Medicine*.

[14] Contessa J., Ciardiello K. A., Perlman S. (2005). Surgery Resident Learning Styles and Academic Achievement. *Current surgery*.

[15] Fowler P. (2002). Learning styles of radiographers. *Radiography*.

[16] Amini N., Zamani BE., Abedini Y. (2010). Medical students' learning styles. *Iran J Med Educ*.

[17] Hosseini L., Seif A. (2001). Learning style's students with regard to sex, sections and educational methods. Seasonal Res Program High Educ. *Learning style's students with regard to sex*.

[18] Jannat Alipour ZNN., Jahanshahi M. (2013). Evaluation of nursing students' learning styles based on VARK learning pattern in. *Ramsar School of Nursing Midwifery. Biannual J Med Edu*.

[19] Javadinia A., Sharifzade G., Abedini M., Khalesi M., Erfaniyan M. (2012). Learning styles of medical students in Birjand University of medical sciences according to VARK model. *Iran J Med Educ*.

[20] Mohammadi S., Mobarhan M., Mohammadi M., Ferns G. (2015). Age and Gender as Determinants of Learning Style among Medical Students. *British Journal of medicine and Medical Research*.

[21] Sarchami R., Hossaini S. (2004). Relationship of learning styles with educational progress of nursing students in Qazvin. *Qazvin Univ Med Sci*.

[22] Valizadeh L., Zamanzadeh V. (2006). Nursing and midwifery students learning styles in Tabriz medical university. *Iran J Med Educ*.

[23] Allaa M., Mirzadeh M., Gharib M., Reza Baradaran H., Khashayar P. (2013). Assessing learning styles of the medical students and faculty in pre-clinical stage of medical education at Tehran University of Medical Sciences. *Zanjan Med Educ J*.

[24] Ghazivakili Z., Norouzi Nia R., Panahi F., Karimi M., Gholsorkhi H., Ahmadi Z. (2014). The role of critical thinking skills and learning styles of university students in their academic performance. *J Adv Med Educ Prof*.

[25] Kalbasi S., Naseri M., Sharifzadeh G., Poursafar A. (2008). Medical students learning styles in. *Poursafar A. Medical students’ learning styles in Birjand University of Medical Sciences. Strides Dev Med Educ*.

[26] Meyari A., Sabouri KA., Gharib M. (2009). Comparison between the learning style of medical freshmen and fifth-year students and its relationship with their educational achievement. Dev Med Educ. *Strides Dev Med Educ*.

[27] Hosseini- Lorgani M., Seif AA. (2001). To compare students' learning styles according to gender, educational grade and major. *Q J Re Plan High Educ*.

[28] Shafian H., Azizzadeh FM., Garrusi B., Haghdoost A. (2014). Predictors of learning styles in students of. *Predictors of learning styles in students of Kerman University of Medical Sciences*.

[29] Baker JD., Reines HD., Wallace CT. (1985). Learning style analysis in surgical training. Am Surg. *Wallace CT. Learning style analysis in surgical training. Am Surg*.

[30] Caulley L., Wadey V., Freeman R. (2012). Learning Styles of First-Year Orthopedic Surgical Residents at 1 Accredited Institution. *Journal of Surgical Education*.

[31] Engels P. T., de Gara C. (2010). Learning styles of medical students, general surgery residents, and general surgeons: implications for surgical education. *BMC Medical Education*.

[32] Laeeq K., Weatherly R. A., Carrott A., Pandian V., Cummings C. W., Bhatti N. I. (2009). Learning styles in two otolaryngology residency programs. *The Laryngoscope*.

[33] Richard R. D., Deegan B. F., Klena J. C. (2014). The Learning Styles of Orthopedic Residents, Faculty, and Applicants at an Academic Program. *Journal of Surgical Education*.

[34] Tuli S. Y., Thompson L. A., Saliba H., Black E. W., Ryan K. A., Kelly M. N., Novak M., Mellott J., Tuli S. S. (2011). Pediatric Residents' Learning Styles and Temperaments and Their Relationships to Standardized Test Scores. *Journal of Graduate Medical Education*.

[35] Modi N., Williams O., Swampillai A. J., Waqar S., Park J., Kersey T. L., Sleep T. (2015). Learning styles and the prospective ophthalmologist. *Medical Teacher*.

[36] Stagg BC., Jensen J., Jorgensen A., Olsen C., Pettey J. (2015). Learning styles among ophthalmology residents. Invest Ophthalmol Vis Sci. *Pettey J. Learning styles among ophthalmology residents. Invest Ophthalmol Vis Sci*.

[37] Kolb A. (2013). *The Kolb Learning Style Inventory 4.0: a comprehensive guide to the theory, psychometrics, research on validity and educational applications*.

[38] Hashemi H., Babamahmoodi A., Fotouhi A., Asgari S. (2015). Revisiting the cataract surgery curriculum for ophthalmology residents: A narrative review. *Iranian Journal of Ophthalmology*.

[39] van den Berg H. (2015). Changes in learning styles induced by practical training. *Learning and Individual Differences*.

[40] Nasiri Z., Gharekhani S., Ghasempour M. (2016). Relationship between Learning Style and Academic Status of Babol Dental Students. *Electronic Physician*.

[41] Salimi M., Sadeghifar J., Peyman H., Shams L. (2012). aural, read/write, and kinesthetic learning styles preferences in students of Isfahan university of medical sciences, Iran. Syst Res. *Iran.  Health Syst Res*.

[42] Sarabi-Asiabar A., Jafari M., Sadeghifar J., Tofighi S., Zaboli R., Peyman H., Salimi M., Shams L. (2014). The Relationship Between Learning Style Preferences and Gender, Educational Major and Status in First Year Medical Students: A Survey Study From Iran. *Iranian Red Crescent Medical Journal*.

[43] Zamani N., Kaboodi A. (2017). Evaluation of the VARK model learning styles selection in medical students. Health Res. *Kaboodi A. Evaluation of the VARK model learning styles selection in medical students. Health Res*.

[44] Shah K., Ahmed J., Shenoy N., Srikant N. (2017). How different are students and their learning styles?. *Int J Res Med Sci*.

[45] Joy S., Kolb D. A. (2009). Are there cultural differences in learning style?. *International Journal of Intercultural Relations*.

